# (2-Amino­phen­yl)methyl­diphenyl­phospho­nium iodide

**DOI:** 10.1107/S160053680803047X

**Published:** 2008-09-27

**Authors:** Zhongshui Li, Meipin Zhu, Aike Li, Jianxin Chen, Li Zhang

**Affiliations:** a College of Chemistry and Materials Science, Fujian Normal University, Fuzhou, Fujian 350007, People’s Republic of China

## Abstract

The asymmetric unit of the title compound, C_19_H_19_NP^+^·I^−^, contains two tetra­alkyl­phospho­nium cations and two I^−^ anions. The P atoms are four-coordinated in distorted tetra­hedral configurations by three phenyl and one methyl C atoms. There are weak intra- and inter­molecular N—H⋯I contacts.

## Related literature

For general background, see: Speiser *et al.* (2005 ▶); Cooper & Downes (1981 ▶); Organ *et al.* (1984 ▶); Wang & Jin (2005 ▶). For related structures, see: Cooper *et al.* (1992 ▶); Li *et al.* (2007 ▶); Zhang *et al.* (2007 ▶).
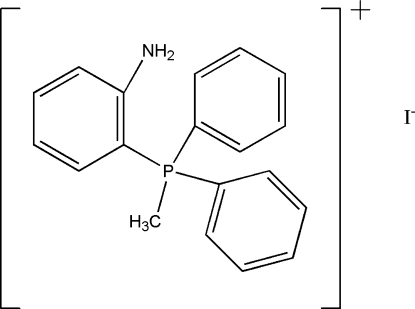


## Experimental

### 

#### Crystal data

C_19_H_19_NP^+^·I^−^
*M* *_r_* = 419.22
Monoclinic, 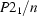

*a* = 13.215 (4) Å
*b* = 17.854 (4) Å
*c* = 15.325 (6) Å
β = 93.385 (14)°
*V* = 3610 (2) Å^3^
*Z* = 8
Mo *K*α radiation
μ = 1.86 mm^−1^
*T* = 298 (2) K
0.50 × 0.40 × 0.40 mm

#### Data collection

Rigaku Weissenberg IP diffractometer
Absorption correction: multi-scan (*TEXSAN*; Molecular Structure Corporation, 1998 ▶) *T*_min_ = 0.420, *T*_max_ = 0.475
32748 measured reflections
8154 independent reflections
6307 reflections with *I* > 2σ(*I*)
*R*_int_ = 0.042

#### Refinement

*R*[*F* ^2^ > 2σ(*F* ^2^)] = 0.030
*wR*(*F* ^2^) = 0.071
*S* = 1.03
8154 reflections
399 parameters
H-atom parameters constrained
Δρ_max_ = 0.70 e Å^−3^
Δρ_min_ = −0.66 e Å^−3^

### 

Data collection: *TEXSAN* (Molecular Structure Corporation, 1998 ▶); cell refinement: *TEXSAN*; data reduction: *TEXSAN*; program(s) used to solve structure: *SHELXS97* (Sheldrick, 2008 ▶); program(s) used to refine structure: *SHELXL97* (Sheldrick, 2008 ▶); molecular graphics: *SHELXTL* (Sheldrick, 2008 ▶) and *PLATON* (Spek, 2003 ▶); software used to prepare material for publication: *SHELXL97*.

## Supplementary Material

Crystal structure: contains datablocks I, global. DOI: 10.1107/S160053680803047X/hk2515sup1.cif

Structure factors: contains datablocks I. DOI: 10.1107/S160053680803047X/hk2515Isup2.hkl

Additional supplementary materials:  crystallographic information; 3D view; checkCIF report

## Figures and Tables

**Table 1:** Hydrogen-bond geometry (Å, °)

*D*—H⋯*A*	*D*—H	H⋯*A*	*D*⋯*A*	*D*—H⋯*A*
N1—H1*A*⋯I2^i^	0.86	2.95	3.773 (3)	160
N2—H2*C*⋯I1	0.86	2.85	3.704 (3)	174

Symmetry code: (i) 
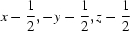
.

**Fig. 1.**
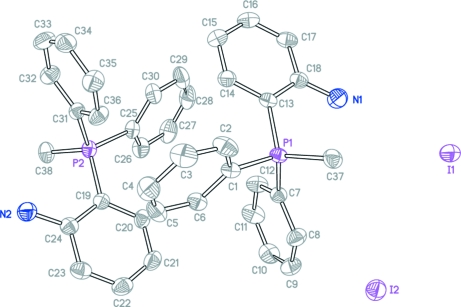
The molecular structure of the title molecule, with the atom-numbering scheme. Displacement ellipsoids are drawn at the 50% probability level.

**Fig. 2.**
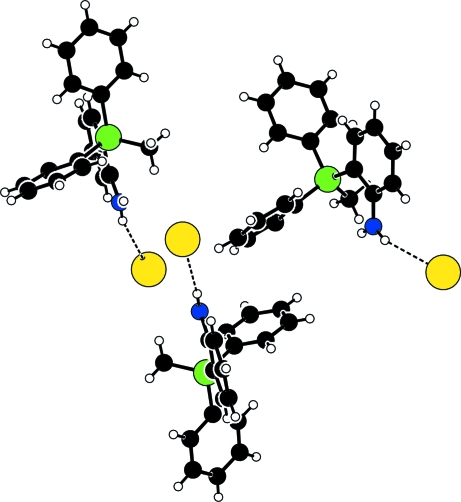
A partial packing diagram of the title compound.

## supplementary crystallographic information

### Comment

The hybrid bidentate phosphine 2-aminophenyldiphenylphosphine was shown to be both a versatile ligand (Cooper *et al.*,  1992; Li *et al.*, 2007; Speiser *et al.*,  2005), forming amino and amido (deprotonated amino) complexes with the later transition metals (Cooper & Downes, 1981; Organ *et al.*, 1984; Wang & Jin, 2005; Zhang *et al.*,  2007), and a useful precursor to novel polydenate and macrocyclic ligands (Cooper *et al.*,  1992). During the reaction between 2-aminophenyldiphenylphosphine and 2-benzoyl-N-methylimidazole to prepare imine complexes, the title compound was obtained unexpectedly, and we report herein its crystal structure.

The asymmetric unit of the title compound (Fig. 1) contains two tetraalkylphosphonium cations and two I^-^ anions. The P atoms are four-coordinated in distorted tetrahedral configurations (Table 1) by three phenyl and one methyl C atoms. There is a weak intramolecular N—H···I contact (Table 2).

In the crystal structure, the weak intermolecular N—H···I contacts (Table 2 and Fig. 2) may be effective in the stabilization of the structure.

### Experimental

The title compound was obtained unexpectedly by the following procedure: 2-aminophenyltriphenylphosphium (8.9 mmol) and 2-benzoyl-N-methylimidazole (8.9 mmol) were dissolved in dry methanol (30 ml) contained in a two necked round bottom flask (50 ml), an oil bubber connected to a nitrogen source, and a magnetic stir bar. A few drops of acetic acid was added. After refluxing for 8 h, solvent removal left a viscous oil. The oil was dissolved in ethanol. The resulting yellow single crystals suitable for X-ray analysis were collected by filtration.

### Refinement

H atoms were positioned geometrically, with N—H = 0.86Å (for NH_2_) and C—H = 0.93 and 0.96Å for aromatic and methyl H atoms, respectively, and constrained to ride on their parent atoms with U_iso_(H) = xU_eq_(C,N), where x = 1.5 for methyl H and x = 1.2 for all other H atoms.

### Crystal data

**Table:**

C_19_H_19_NP^+^·I^−^	*F*(000) = 1664
*M**_r_* = 419.22	*D*_x_ = 1.543 Mg m^−^^3^*D*_m_ = no Mg m^−^^3^*D*_m_ measured by not measured
Monoclinic, *P*2_1_/*n*	Melting point: no K
Hall symbol: -P 2yn	Mo *K*α radiation, λ = 0.71073 Å
*a* = 13.215 (4) Å	Cell parameters from 8154 reflections
*b* = 17.854 (4) Å	θ = 3.0–27.5°
*c* = 15.325 (6) Å	µ = 1.86 mm^−^^1^
β = 93.385 (14)°	*T* = 298 K
*V* = 3610 (2) Å^3^	Block, yellow
*Z* = 8	0.50 × 0.40 × 0.40 mm

### Data collection

**Table:**

Rigaku Weissenberg IP diffractometer	8154 independent reflections
Radiation source: fine-focus sealed tube	6307 reflections with *I* > 2σ(*I*)
graphite	*R*_int_ = 0.042
Detector resolution: no pixels mm^-1^	θ_max_ = 27.5°, θ_min_ = 3.0°
scintillation counter scans	*h* = −17→17
Absorption correction: multi-scan (TEXSAN; Molecular Structure Corporation, 1998)	*k* = −22→23
*T*_min_ = 0.420, *T*_max_ = 0.475	*l* = −19→19
32748 measured reflections	

### Refinement

**Table:**

Refinement on *F*^2^	Primary atom site location: structure-invariant direct methods
Least-squares matrix: full	Secondary atom site location: difference Fourier map
*R*[*F*^2^ > 2σ(*F*^2^)] = 0.030	Hydrogen site location: inferred from neighbouring sites
*wR*(*F*^2^) = 0.071	H-atom parameters constrained
*S* = 1.03	*w* = 1/[σ^2^(*F*_o_^2^) + (0.031*P*)^2^] where *P* = (*F*_o_^2^ + 2*F*_c_^2^)/3
8154 reflections	(Δ/σ)_max_ = 0.001
399 parameters	Δρ_max_ = 0.70 e Å^−^^3^
0 restraints	Δρ_min_ = −0.66 e Å^−^^3^

### Special details

**Table:**

Geometry. All e.s.d.'s (except the e.s.d. in the dihedral angle between two l.s. planes) are estimated using the full covariance matrix. The cell e.s.d.'s are taken into account individually in the estimation of e.s.d.'s in distances, angles and torsion angles; correlations between e.s.d.'s in cell parameters are only used when they are defined by crystal symmetry. An approximate (isotropic) treatment of cell e.s.d.'s is used for estimating e.s.d.'s involving l.s. planes.
Refinement. Refinement of *F*^2^ against ALL reflections. The weighted *R*-factor *wR* and goodness of fit *S* are based on *F*^2^, conventional *R*-factors *R* are based on *F*, with *F* set to zero for negative *F*^2^. The threshold expression of *F*^2^ > σ(*F*^2^) is used only for calculating *R*-factors(gt) *etc*. and is not relevant to the choice of reflections for refinement. *R*-factors based on *F*^2^ are statistically about twice as large as those based on *F*, and *R*- factors based on ALL data will be even larger.

### Fractional atomic coordinates and isotropic or equivalent isotropic displacement parameters (Å^2^)

**Table:**

	*x*	*y*	*z*	*U*_iso_*/*U*_eq_	
I1	0.275253 (16)	−0.173440 (9)	0.146444 (14)	0.06249 (7)	
I2	0.246225 (14)	0.039910 (9)	0.389305 (12)	0.05489 (6)	
P1	0.21547 (4)	−0.23074 (3)	0.46752 (4)	0.03795 (13)	
P2	0.34957 (4)	−0.41826 (3)	0.81596 (4)	0.04135 (14)	
N1	0.2607 (2)	−0.33502 (12)	0.30937 (18)	0.0668 (7)	
H1A	0.2768	−0.3620	0.2658	0.080*	
H1B	0.2769	−0.2884	0.3115	0.080*	
N2	0.30679 (19)	−0.30209 (13)	0.96039 (17)	0.0629 (6)	
H2B	0.2868	−0.3479	0.9614	0.076*	
H2C	0.2939	−0.2723	1.0024	0.076*	
C1	0.16106 (17)	−0.20039 (12)	0.56607 (16)	0.0413 (5)	
C2	0.0564 (2)	−0.19164 (16)	0.5673 (2)	0.0602 (8)	
H2A	0.0159	−0.1986	0.5163	0.072*	
C3	0.0128 (2)	−0.17288 (16)	0.6432 (2)	0.0692 (9)	
H3A	−0.0571	−0.1671	0.6435	0.083*	
C4	0.0720 (3)	−0.16268 (16)	0.7185 (2)	0.0676 (8)	
H4A	0.0422	−0.1507	0.7701	0.081*	
C5	0.1752 (3)	−0.17011 (15)	0.7179 (2)	0.0611 (7)	
H5A	0.2153	−0.1627	0.7691	0.073*	
C6	0.2199 (2)	−0.18849 (12)	0.64174 (18)	0.0471 (6)	
H6A	0.2900	−0.1929	0.6416	0.056*	
C7	0.34992 (17)	−0.22050 (12)	0.48204 (16)	0.0417 (5)	
C8	0.3946 (2)	−0.15029 (15)	0.4743 (2)	0.0574 (7)	
H8A	0.3558	−0.1097	0.4541	0.069*	
C9	0.4958 (2)	−0.14136 (17)	0.4966 (2)	0.0677 (8)	
H9A	0.5257	−0.0945	0.4924	0.081*	
C10	0.5530 (2)	−0.20159 (19)	0.5252 (2)	0.0661 (8)	
H10A	0.6216	−0.1951	0.5406	0.079*	
C11	0.5107 (2)	−0.27100 (17)	0.5314 (2)	0.0667 (8)	
H11A	0.5506	−0.3115	0.5501	0.080*	
C12	0.40877 (19)	−0.28112 (14)	0.5101 (2)	0.0555 (7)	
H12A	0.3797	−0.3283	0.5144	0.067*	
C13	0.18399 (17)	−0.32710 (11)	0.45066 (16)	0.0399 (5)	
C14	0.13475 (19)	−0.36486 (13)	0.51540 (18)	0.0474 (6)	
H14A	0.1178	−0.3391	0.5652	0.057*	
C15	0.1108 (2)	−0.43940 (14)	0.5070 (2)	0.0559 (7)	
H15A	0.0775	−0.4639	0.5506	0.067*	
C16	0.1366 (2)	−0.47742 (14)	0.4334 (2)	0.0570 (7)	
H16A	0.1208	−0.5280	0.4277	0.068*	
C17	0.18451 (19)	−0.44243 (13)	0.3692 (2)	0.0515 (6)	
H17A	0.2011	−0.4696	0.3203	0.062*	
C18	0.20976 (18)	−0.36601 (13)	0.37475 (17)	0.0456 (6)	
C19	0.38306 (17)	−0.32116 (12)	0.82121 (17)	0.0429 (5)	
C20	0.43743 (19)	−0.28979 (15)	0.75442 (18)	0.0503 (6)	
H20A	0.4531	−0.3191	0.7069	0.060*	
C21	0.4678 (2)	−0.21594 (16)	0.7585 (2)	0.0596 (7)	
H21A	0.5032	−0.1953	0.7137	0.071*	
C22	0.4457 (2)	−0.17343 (16)	0.8287 (2)	0.0629 (8)	
H22A	0.4676	−0.1240	0.8318	0.076*	
C23	0.3919 (2)	−0.20197 (15)	0.8946 (2)	0.0587 (7)	
H23A	0.3772	−0.1714	0.9413	0.070*	
C24	0.35822 (18)	−0.27696 (13)	0.89301 (18)	0.0484 (6)	
C25	0.39532 (19)	−0.46171 (13)	0.72040 (17)	0.0470 (6)	
C26	0.4990 (2)	−0.47327 (17)	0.7155 (2)	0.0623 (7)	
H26A	0.5445	−0.4541	0.7585	0.075*	
C27	0.5336 (3)	−0.5131 (2)	0.6468 (3)	0.0788 (10)	
H27A	0.6028	−0.5203	0.6427	0.095*	
C28	0.4667 (3)	−0.54243 (18)	0.5843 (2)	0.0809 (10)	
H28A	0.4910	−0.5706	0.5389	0.097*	
C29	0.3650 (3)	−0.53088 (19)	0.5878 (2)	0.0769 (9)	
H29A	0.3202	−0.5507	0.5447	0.092*	
C30	0.3288 (2)	−0.48961 (16)	0.65574 (19)	0.0590 (7)	
H30A	0.2596	−0.4807	0.6578	0.071*	
C31	0.21474 (18)	−0.42786 (12)	0.81176 (16)	0.0421 (5)	
C32	0.1681 (2)	−0.49125 (14)	0.8414 (2)	0.0582 (7)	
H32A	0.2064	−0.5284	0.8703	0.070*	
C33	0.0645 (2)	−0.49951 (17)	0.8283 (2)	0.0694 (8)	
H33A	0.0333	−0.5427	0.8475	0.083*	
C34	0.0070 (2)	−0.44431 (18)	0.7870 (2)	0.0650 (8)	
H34A	−0.0627	−0.4503	0.7782	0.078*	
C35	0.0522 (2)	−0.38083 (17)	0.7588 (2)	0.0637 (8)	
H35A	0.0131	−0.3435	0.7313	0.076*	
C36	0.15576 (19)	−0.37169 (15)	0.7709 (2)	0.0543 (7)	
H36A	0.1862	−0.3282	0.7519	0.065*	
C37	0.1658 (2)	−0.17182 (13)	0.38091 (19)	0.0541 (7)	
H37A	0.1934	−0.1867	0.3270	0.081*	
H37B	0.1839	−0.1207	0.3935	0.081*	
H37C	0.0933	−0.1763	0.3757	0.081*	
C38	0.4077 (2)	−0.46872 (15)	0.90723 (18)	0.0568 (7)	
H38A	0.3858	−0.4478	0.9607	0.085*	
H38B	0.4801	−0.4648	0.9064	0.085*	
H38C	0.3881	−0.5205	0.9033	0.085*	

### Atomic displacement parameters (Å^2^)

**Table:**

	*U*^11^	*U*^22^	*U*^33^	*U*^12^	*U*^13^	*U*^23^
I1	0.06953 (13)	0.05555 (10)	0.06229 (14)	−0.01435 (9)	0.00288 (9)	−0.00578 (8)
I2	0.06649 (12)	0.04646 (9)	0.05246 (12)	0.00345 (8)	0.00974 (8)	−0.00069 (7)
P1	0.0329 (3)	0.0367 (3)	0.0434 (4)	−0.0001 (2)	−0.0050 (2)	−0.0038 (2)
P2	0.0374 (3)	0.0479 (3)	0.0388 (4)	0.0053 (3)	0.0025 (2)	0.0052 (2)
N1	0.0872 (18)	0.0564 (12)	0.0591 (17)	−0.0070 (12)	0.0244 (13)	−0.0138 (11)
N2	0.0675 (15)	0.0593 (12)	0.0638 (17)	−0.0099 (12)	0.0195 (12)	−0.0092 (11)
C1	0.0361 (11)	0.0373 (10)	0.0499 (15)	−0.0006 (10)	−0.0013 (10)	−0.0056 (9)
C2	0.0374 (13)	0.0737 (17)	0.069 (2)	0.0038 (13)	−0.0052 (13)	−0.0219 (14)
C3	0.0462 (15)	0.0749 (18)	0.088 (3)	0.0090 (14)	0.0137 (16)	−0.0209 (16)
C4	0.078 (2)	0.0615 (16)	0.065 (2)	0.0071 (16)	0.0250 (17)	−0.0126 (14)
C5	0.076 (2)	0.0607 (15)	0.0457 (17)	0.0054 (14)	−0.0034 (14)	−0.0122 (12)
C6	0.0444 (13)	0.0442 (12)	0.0517 (17)	0.0042 (11)	−0.0056 (11)	−0.0036 (10)
C7	0.0346 (11)	0.0467 (11)	0.0435 (14)	−0.0018 (10)	0.0008 (10)	−0.0023 (9)
C8	0.0474 (14)	0.0558 (14)	0.068 (2)	−0.0087 (13)	−0.0006 (13)	0.0111 (13)
C9	0.0540 (16)	0.0747 (19)	0.075 (2)	−0.0247 (16)	0.0072 (15)	0.0050 (15)
C10	0.0337 (12)	0.094 (2)	0.070 (2)	−0.0050 (15)	0.0034 (13)	−0.0072 (17)
C11	0.0416 (14)	0.0733 (18)	0.084 (2)	0.0136 (14)	−0.0080 (14)	−0.0139 (15)
C12	0.0400 (13)	0.0503 (13)	0.075 (2)	0.0041 (11)	−0.0053 (13)	−0.0077 (12)
C13	0.0356 (11)	0.0383 (10)	0.0453 (14)	−0.0005 (9)	−0.0036 (10)	−0.0058 (9)
C14	0.0469 (13)	0.0463 (12)	0.0490 (16)	−0.0050 (11)	0.0035 (11)	−0.0042 (10)
C15	0.0534 (15)	0.0488 (13)	0.066 (2)	−0.0086 (12)	0.0069 (13)	0.0023 (12)
C16	0.0489 (14)	0.0421 (12)	0.080 (2)	−0.0055 (12)	0.0009 (14)	−0.0082 (12)
C17	0.0431 (13)	0.0463 (12)	0.0649 (19)	0.0016 (11)	0.0016 (12)	−0.0159 (11)
C18	0.0394 (12)	0.0482 (12)	0.0491 (16)	0.0002 (10)	0.0021 (11)	−0.0070 (10)
C19	0.0329 (11)	0.0505 (12)	0.0445 (15)	−0.0007 (10)	−0.0035 (10)	0.0040 (10)
C20	0.0449 (13)	0.0605 (14)	0.0449 (16)	0.0013 (12)	−0.0026 (11)	0.0120 (11)
C21	0.0510 (15)	0.0682 (16)	0.0584 (19)	−0.0098 (14)	−0.0054 (13)	0.0220 (14)
C22	0.0557 (16)	0.0597 (15)	0.071 (2)	−0.0132 (13)	−0.0152 (15)	0.0127 (14)
C23	0.0549 (15)	0.0541 (14)	0.065 (2)	−0.0018 (13)	−0.0098 (14)	−0.0042 (13)
C24	0.0390 (12)	0.0532 (13)	0.0522 (17)	−0.0007 (11)	−0.0032 (11)	0.0012 (11)
C25	0.0475 (13)	0.0523 (12)	0.0418 (15)	0.0085 (11)	0.0080 (11)	0.0064 (10)
C26	0.0478 (15)	0.0783 (18)	0.062 (2)	0.0108 (14)	0.0118 (13)	0.0048 (14)
C27	0.069 (2)	0.094 (2)	0.076 (3)	0.0229 (19)	0.0317 (19)	0.0120 (19)
C28	0.112 (3)	0.076 (2)	0.059 (2)	0.024 (2)	0.037 (2)	0.0026 (16)
C29	0.099 (3)	0.088 (2)	0.0444 (19)	0.006 (2)	0.0065 (17)	−0.0067 (15)
C30	0.0576 (16)	0.0761 (17)	0.0433 (17)	0.0080 (14)	0.0033 (13)	0.0010 (13)
C31	0.0402 (12)	0.0436 (11)	0.0432 (15)	0.0006 (10)	0.0093 (10)	−0.0015 (9)
C32	0.0593 (16)	0.0474 (13)	0.069 (2)	−0.0003 (13)	0.0111 (14)	0.0024 (12)
C33	0.0682 (19)	0.0611 (16)	0.081 (2)	−0.0247 (16)	0.0187 (17)	−0.0029 (15)
C34	0.0441 (14)	0.086 (2)	0.066 (2)	−0.0122 (15)	0.0065 (14)	−0.0136 (15)
C35	0.0416 (14)	0.0784 (18)	0.070 (2)	0.0025 (14)	−0.0012 (13)	0.0112 (15)
C36	0.0400 (13)	0.0558 (14)	0.0669 (19)	−0.0021 (11)	0.0005 (12)	0.0163 (12)
C37	0.0595 (16)	0.0490 (13)	0.0519 (17)	0.0025 (12)	−0.0116 (13)	0.0024 (11)
C38	0.0585 (16)	0.0671 (15)	0.0443 (16)	0.0171 (13)	−0.0013 (12)	0.0104 (12)

### Geometric parameters (Å, °)

**Table:**

P1—C13	1.785 (2)	C16—C17	1.355 (4)
P1—C7	1.787 (2)	C16—H16A	0.9300
P1—C37	1.788 (3)	C17—C18	1.406 (3)
P1—C1	1.794 (3)	C17—H17A	0.9300
P2—C31	1.787 (2)	C19—C20	1.402 (4)
P2—C19	1.790 (2)	C19—C24	1.409 (4)
P2—C25	1.793 (3)	C20—C21	1.379 (4)
P2—C38	1.797 (3)	C20—H20A	0.9300
N1—C18	1.358 (4)	C21—C22	1.362 (5)
N1—H1A	0.8600	C21—H21A	0.9300
N1—H1B	0.8600	C22—C23	1.368 (5)
N2—C24	1.346 (4)	C22—H22A	0.9300
N2—H2B	0.8600	C23—C24	1.411 (4)
N2—H2C	0.8600	C23—H23A	0.9300
C1—C6	1.374 (3)	C25—C30	1.378 (4)
C1—C2	1.393 (3)	C25—C26	1.392 (4)
C2—C3	1.369 (5)	C26—C27	1.371 (5)
C2—H2A	0.9300	C26—H26A	0.9300
C3—C4	1.367 (5)	C27—C28	1.368 (5)
C3—H3A	0.9300	C27—H27A	0.9300
C4—C5	1.371 (4)	C28—C29	1.364 (5)
C4—H4A	0.9300	C28—H28A	0.9300
C5—C6	1.378 (4)	C29—C30	1.384 (4)
C5—H5A	0.9300	C29—H29A	0.9300
C6—H6A	0.9300	C30—H30A	0.9300
C7—C12	1.386 (3)	C31—C32	1.379 (3)
C7—C8	1.393 (3)	C31—C36	1.395 (3)
C8—C9	1.370 (4)	C32—C33	1.380 (4)
C8—H8A	0.9300	C32—H32A	0.9300
C9—C10	1.372 (4)	C33—C34	1.376 (4)
C9—H9A	0.9300	C33—H33A	0.9300
C10—C11	1.365 (4)	C34—C35	1.363 (4)
C10—H10A	0.9300	C34—H34A	0.9300
C11—C12	1.379 (4)	C35—C36	1.379 (4)
C11—H11A	0.9300	C35—H35A	0.9300
C12—H12A	0.9300	C36—H36A	0.9300
C13—C14	1.392 (4)	C37—H37A	0.9600
C13—C18	1.414 (3)	C37—H37B	0.9600
C14—C15	1.372 (3)	C37—H37C	0.9600
C14—H14A	0.9300	C38—H38A	0.9600
C15—C16	1.377 (4)	C38—H38B	0.9600
C15—H15A	0.9300	C38—H38C	0.9600
			
C13—P1—C7	109.76 (10)	N1—C18—C17	118.5 (2)
C13—P1—C37	112.79 (11)	N1—C18—C13	124.1 (2)
C7—P1—C37	110.56 (13)	C17—C18—C13	117.3 (2)
C13—P1—C1	108.14 (12)	C20—C19—C24	120.0 (2)
C7—P1—C1	108.01 (11)	C20—C19—P2	119.4 (2)
C37—P1—C1	107.41 (13)	C24—C19—P2	120.6 (2)
C31—P2—C19	109.75 (10)	C21—C20—C19	120.6 (3)
C31—P2—C25	108.21 (12)	C21—C20—H20A	119.7
C19—P2—C25	111.18 (12)	C19—C20—H20A	119.7
C31—P2—C38	111.04 (13)	C22—C21—C20	119.6 (3)
C19—P2—C38	110.94 (12)	C22—C21—H21A	120.2
C25—P2—C38	105.63 (13)	C20—C21—H21A	120.2
C18—N1—H1A	120.0	C21—C22—C23	121.4 (3)
C18—N1—H1B	120.0	C21—C22—H22A	119.3
H1A—N1—H1B	120.0	C23—C22—H22A	119.3
C24—N2—H2B	120.0	C22—C23—C24	121.2 (3)
C24—N2—H2C	120.0	C22—C23—H23A	119.4
H2B—N2—H2C	120.0	C24—C23—H23A	119.4
C6—C1—C2	118.9 (2)	N2—C24—C19	124.3 (2)
C6—C1—P1	121.49 (19)	N2—C24—C23	118.4 (3)
C2—C1—P1	119.55 (19)	C19—C24—C23	117.2 (3)
C3—C2—C1	120.5 (3)	C30—C25—C26	119.7 (3)
C3—C2—H2A	119.8	C30—C25—P2	120.8 (2)
C1—C2—H2A	119.8	C26—C25—P2	119.2 (2)
C4—C3—C2	120.1 (3)	C27—C26—C25	119.4 (3)
C4—C3—H3A	120.0	C27—C26—H26A	120.3
C2—C3—H3A	120.0	C25—C26—H26A	120.3
C3—C4—C5	120.1 (3)	C28—C27—C26	120.4 (3)
C3—C4—H4A	120.0	C28—C27—H27A	119.8
C5—C4—H4A	120.0	C26—C27—H27A	119.8
C4—C5—C6	120.3 (3)	C29—C28—C27	120.8 (3)
C4—C5—H5A	119.9	C29—C28—H28A	119.6
C6—C5—H5A	119.9	C27—C28—H28A	119.6
C1—C6—C5	120.2 (3)	C28—C29—C30	119.7 (3)
C1—C6—H6A	119.9	C28—C29—H29A	120.2
C5—C6—H6A	119.9	C30—C29—H29A	120.2
C12—C7—C8	119.7 (2)	C25—C30—C29	120.0 (3)
C12—C7—P1	119.62 (18)	C25—C30—H30A	120.0
C8—C7—P1	120.29 (18)	C29—C30—H30A	120.0
C9—C8—C7	119.7 (3)	C32—C31—C36	119.3 (2)
C9—C8—H8A	120.2	C32—C31—P2	122.06 (19)
C7—C8—H8A	120.2	C36—C31—P2	118.45 (18)
C8—C9—C10	120.0 (3)	C31—C32—C33	119.9 (3)
C8—C9—H9A	120.0	C31—C32—H32A	120.1
C10—C9—H9A	120.0	C33—C32—H32A	120.1
C11—C10—C9	120.9 (2)	C34—C33—C32	120.5 (3)
C11—C10—H10A	119.6	C34—C33—H33A	119.8
C9—C10—H10A	119.6	C32—C33—H33A	119.8
C10—C11—C12	120.1 (3)	C35—C34—C33	120.1 (3)
C10—C11—H11A	120.0	C35—C34—H34A	120.0
C12—C11—H11A	120.0	C33—C34—H34A	120.0
C11—C12—C7	119.6 (2)	C34—C35—C36	120.4 (3)
C11—C12—H12A	120.2	C34—C35—H35A	119.8
C7—C12—H12A	120.2	C36—C35—H35A	119.8
C14—C13—C18	119.6 (2)	C35—C36—C31	119.9 (2)
C14—C13—P1	118.57 (18)	C35—C36—H36A	120.1
C18—C13—P1	121.82 (19)	C31—C36—H36A	120.1
C15—C14—C13	121.2 (2)	P1—C37—H37A	109.5
C15—C14—H14A	119.4	P1—C37—H37B	109.5
C13—C14—H14A	119.4	H37A—C37—H37B	109.5
C14—C15—C16	119.2 (3)	P1—C37—H37C	109.5
C14—C15—H15A	120.4	H37A—C37—H37C	109.5
C16—C15—H15A	120.4	H37B—C37—H37C	109.5
C17—C16—C15	121.2 (2)	P2—C38—H38A	109.5
C17—C16—H16A	119.4	P2—C38—H38B	109.5
C15—C16—H16A	119.4	H38A—C38—H38B	109.5
C16—C17—C18	121.5 (3)	P2—C38—H38C	109.5
C16—C17—H17A	119.2	H38A—C38—H38C	109.5
C18—C17—H17A	119.2	H38B—C38—H38C	109.5
			
C13—P1—C1—C6	106.0 (2)	C31—P2—C19—C20	−119.13 (19)
C7—P1—C1—C6	−12.7 (2)	C25—P2—C19—C20	0.6 (2)
C37—P1—C1—C6	−132.0 (2)	C38—P2—C19—C20	117.8 (2)
C13—P1—C1—C2	−71.2 (2)	C31—P2—C19—C24	63.0 (2)
C7—P1—C1—C2	170.1 (2)	C25—P2—C19—C24	−177.35 (18)
C37—P1—C1—C2	50.8 (2)	C38—P2—C19—C24	−60.1 (2)
C6—C1—C2—C3	−1.2 (4)	C24—C19—C20—C21	0.6 (3)
P1—C1—C2—C3	176.1 (2)	P2—C19—C20—C21	−177.34 (19)
C1—C2—C3—C4	−0.1 (5)	C19—C20—C21—C22	0.7 (4)
C2—C3—C4—C5	1.0 (5)	C20—C21—C22—C23	−1.4 (4)
C3—C4—C5—C6	−0.6 (4)	C21—C22—C23—C24	0.8 (4)
C2—C1—C6—C5	1.6 (4)	C20—C19—C24—N2	−179.7 (2)
P1—C1—C6—C5	−175.60 (19)	P2—C19—C24—N2	−1.8 (3)
C4—C5—C6—C1	−0.8 (4)	C20—C19—C24—C23	−1.2 (3)
C13—P1—C7—C12	−23.0 (3)	P2—C19—C24—C23	176.74 (18)
C37—P1—C7—C12	−148.0 (2)	C22—C23—C24—N2	179.1 (2)
C1—P1—C7—C12	94.7 (2)	C22—C23—C24—C19	0.5 (4)
C13—P1—C7—C8	163.8 (2)	C31—P2—C25—C30	5.3 (2)
C37—P1—C7—C8	38.7 (3)	C19—P2—C25—C30	−115.3 (2)
C1—P1—C7—C8	−78.5 (2)	C38—P2—C25—C30	124.2 (2)
C12—C7—C8—C9	−1.7 (4)	C31—P2—C25—C26	−168.9 (2)
P1—C7—C8—C9	171.5 (3)	C19—P2—C25—C26	70.5 (2)
C7—C8—C9—C10	0.9 (5)	C38—P2—C25—C26	−50.0 (2)
C8—C9—C10—C11	0.4 (5)	C30—C25—C26—C27	−0.8 (4)
C9—C10—C11—C12	−0.9 (5)	P2—C25—C26—C27	173.4 (2)
C10—C11—C12—C7	0.2 (5)	C25—C26—C27—C28	−1.1 (5)
C8—C7—C12—C11	1.1 (4)	C26—C27—C28—C29	1.8 (5)
P1—C7—C12—C11	−172.1 (2)	C27—C28—C29—C30	−0.6 (5)
C7—P1—C13—C14	111.5 (2)	C26—C25—C30—C29	2.0 (4)
C37—P1—C13—C14	−124.8 (2)	P2—C25—C30—C29	−172.2 (2)
C1—P1—C13—C14	−6.2 (2)	C28—C29—C30—C25	−1.3 (5)
C7—P1—C13—C18	−67.1 (2)	C19—P2—C31—C32	−153.7 (2)
C37—P1—C13—C18	56.7 (2)	C25—P2—C31—C32	84.8 (2)
C1—P1—C13—C18	175.32 (18)	C38—P2—C31—C32	−30.7 (3)
C18—C13—C14—C15	0.1 (4)	C19—P2—C31—C36	31.3 (3)
P1—C13—C14—C15	−178.4 (2)	C25—P2—C31—C36	−90.1 (2)
C13—C14—C15—C16	0.4 (4)	C38—P2—C31—C36	154.4 (2)
C14—C15—C16—C17	−0.4 (4)	C36—C31—C32—C33	1.9 (4)
C15—C16—C17—C18	−0.1 (4)	P2—C31—C32—C33	−173.0 (2)
C16—C17—C18—N1	177.9 (3)	C31—C32—C33—C34	−1.1 (5)
C16—C17—C18—C13	0.6 (4)	C32—C33—C34—C35	−0.1 (5)
C14—C13—C18—N1	−177.7 (2)	C33—C34—C35—C36	0.5 (5)
P1—C13—C18—N1	0.8 (3)	C34—C35—C36—C31	0.3 (5)
C14—C13—C18—C17	−0.6 (3)	C32—C31—C36—C35	−1.5 (4)
P1—C13—C18—C17	177.91 (17)	P2—C31—C36—C35	173.6 (2)

### Hydrogen-bond geometry (Å, °)

**Table:**

*D*—H···*A*	*D*—H	H···*A*	*D*···*A*	*D*—H···*A*
N1—H1A···I2^i^	0.86	2.95	3.773 (3)	160.
N2—H2C···I1	0.86	2.85	3.704 (3)	174.

Symmetry codes: (i) *x*−1/2, −*y*−1/2, *z*−1/2.
